# Clinical application of artificial neural network (ANN) modeling to predict *BRCA1/2* germline deleterious variants in Chinese bilateral primary breast cancer patients

**DOI:** 10.1186/s12885-022-10160-y

**Published:** 2022-11-02

**Authors:** Yan Li, Lili Chen, Jinxing Lv, Xiaobin Chen, Bangwei Zeng, Minyan Chen, Wenhui Guo, Yuxiang Lin, Liuwen Yu, Jialin Hou, Jing Li, Peng Zhou, Wenzhe Zhang, Shengmei Li, Xuan Jin, Weifeng Cai, Kun Zhang, Yeyuan Huang, Chuan Wang, Fangmeng Fu

**Affiliations:** 1 Department of Breast Surgery, Fujian Medical University Union Hospital, No.29, Xin Quan Road, Gulou District, 350001 Fuzhou, Fujian Province China; 2 Department of General Surgery, Fujian Medical University Union Hospital, 350001 Fuzhou, Fujian Province China; 3 Breast Cancer Institute, Fujian Medical University, 350001 Fuzhou, Fujian Province China; 4 Sichuan Cancer Center, School of Medicine, Sichuan Cancer Hospital & Institute, University of Electronic Science and Technology of China, 610000 Chengdu, China; 5 Nosocomial Infection Control Branch, Fujian Medical University Union Hospital, Fuzhou, Fujian Province China; 6 Fujian Medical University, 350001 Fuzhou, Fujian Province China

**Keywords:** Bilateral breast cancer, *BRCA1*, *BRCA2*, Germline deleterious variant, Artificial neural network

## Abstract

**Background:**

Bilateral breast cancer (BBC), as well as ovarian cancer, are significantly associated with germline deleterious variants in *BRCA1/2*, while *BRCA1/2* germline deleterious variants carriers can exquisitely benefit from poly (ADP-ribose) polymerase (PARP) inhibitors. However, formal genetic testing could not be carried out for all patients due to extensive use of healthcare resources, which in turn results in high medical costs. To date, existing *BRCA1/2* deleterious variants prediction models have been developed in women of European or other descent who are quite genetically different from Asian population. Therefore, there is an urgent clinical need for tools to predict the frequency of *BRCA1/2* deleterious variants in Asian BBC patients balancing the increased demand for and cost of cancer genetics services.

**Methods:**

The entire coding region of *BRCA1/2* was screened for the presence of germline deleterious variants by the next generation sequencing in 123 Chinese BBC patients. Chi-square test, univariate and multivariate logistic regression were used to assess the relationship between *BRCA1/2* germline deleterious variants and clinicopathological characteristics. The R software was utilized to develop artificial neural network (ANN) and nomogram modeling for *BRCA1/2* germline deleterious variants prediction.

**Results:**

Among 123 BBC patients, we identified a total of 20 deleterious variants in *BRCA1* (8; 6.5%) and *BRCA2* (12; 9.8%). c.5485del in *BRCA1* is novel frameshift deleterious variant. Deleterious variants carriers were younger at first diagnosis (*P* = 0.0003), with longer interval between two tumors (*P* = 0.015), at least one medullary carcinoma (*P* = 0.001), and more likely to be hormone receptor negative (*P* = 0.006) and *HER2* negative (*P* = 0.001). Area under the receiver operating characteristic curve was 0.903 in ANN and 0.828 in nomogram modeling individually (*P* = 0.02).

**Conclusion:**

This study shows the spectrum of the *BRCA1/2* germline deleterious variants in Chinese BBC patients and indicates that the ANN can accurately predict BRCA deleterious variants than conventional statistical linear approach, which confirms the *BRCA1/2* deleterious variants carriers at the lowest costs without adding any additional examinations.

**Supplementary information:**

The online version contains supplementary material available at 10.1186/s12885-022-10160-y.

## Background

Bilateral breast cancer (BBC), categorized as synchronous and metachronous disease, is observed in 2-11% of breast cancer cases [1, 2]. Patients with breast cancer have a 2-20% chance of developing a contralateral breast cancer (CBC), either synchronously detected, or as a metachronous cancer [3]. The increased number of breast cancer cases and improved survival after the first BC diagnosis contribute to the current higher incidence of BBC. Several factors are thought to be associated with the occurrence and development of bilateral breast cancer, such as early age at diagnosis, histology, family history, and especially the presence of germline deleterious variants that include *BRCA1/2*, *PALB2*, *CDH1*, and *CHEK2*. Studies have reported that the prognosis of BBC patients is similar or worse than unilateral breast cancer (UBC) patients [4–7].

*BRCA1* and *BRCA2* genes are involved in homologous recombination repair. Germline *BRCA1* and *BRCA2* loss-of-function variants predispose to development of breast cancer. Previous research has demonstrated that the *BRCA1/2* deleterious variant frequency in BBC (29.6%) is significantly higher than the rate in unselected breast cancer (5.4%) [8, 9]. Of note, patients with bilateral breast cancer are suggested by National Comprehensive Cancer Network (NCCN) Guidelines to undergo further genetic risk evaluation [10]. Identifying *BRCA1/2* deleterious variants carriers could not only shed light on adjusting chemotherapy schemes, but also contribute to the prevention of ovarian cancer and offspring onset. Secondary analyses of the GeparOcto and GeparSixto Randomized Clinical Trial have revealed higher pathological complete response (pCR) rates in *BRCA1* and *BRCA2* deleterious variants carriers [11, 12].

However, in low- and middle- income countries, only a small number of studies that included Chinese bilateral breast cancer patients have been reported and related clinical characteristics have not always been well clarified. Thus, taking uncovered health insurance together, Chinese physicians are always cautious over recommending expensive genetic risk evaluation. Many models have been developed for predicting the likelihood of carrying germline BRCA deleterious variants using data of patients with breast cancer. However, these models underestimate *BRCA1/2* deleterious variants carriers and cannot distinguish well between carriers and non-carriers in Asian breast cancer patients [13]. What’s more, although BBC status has been considered as an indicator of BRCA deleterious variants in Asian populations, models are built on data from breast cancer patients, instead of the BBC population [14–16].

In this study, we performed the next generation sequencing for all exons of *BRCA1* and *BRCA2* in 123 Chinese BBC patients to analyze the relationship between *BRCA1/2* germline deleterious variants and characteristics of BBC. We aimed to construct a user-friendly model to predict the risk of *BRCA1/2* deleterious variants in Chinese BBC patients.

## Methods

### Study patients

We conducted a retrospective study of patients diagnosed with BBC who were treated in the Fujian Medical University Union Hospital from 2005 to 2021. In this study BBC was classified as metachronous bilateral breast cancer (MBBC) and synchronous bilateral breast cancer (SBBC), with an interval between the first and contralateral breast cancer of ≥ 2 years and < 2 years, respectively. All patients were histopathologically confirmed by at least two pathologists. Patients with metastasis that occurred before or at the same time as bilateral breast cancer were excluded. Clinicopathological characteristics were obtained after informed consent that included menstrual history, reproductive history, lactation history, family history, ER (estrogen receptor), PR (progesterone receptor), HER2 (human epidermal growth factor receptor 2), and chemotherapy or radiation status. About 5ml of peripheral venous blood was collected individually.

### DNA extraction and sequencing

The genomic DNA was isolated from peripheral blood lymphocytes using Large amount of whole blood genomic DNA extraction kits (DP2202, Bioteke, China) according to the manufacturer’s instructions. DNA purity and concentration were assessed by the NanoDrop2000 spectrophotometer (Thermo Fisher Scientific) and DNA quality was assessed by agarose gel electrophoresis.

Library preparation and sequencing of all coding regions and exon-intron boundaries of the *BRCA1* and *BRCA2* genes were performed through next generation sequencing (Illumina Novaseq) by shanghai aita gene technology Co.Ltd with Human *BRCA1/BRCA2* Gene Mutation Detection Kit. The libraries were quantified by Qubit™ dsDNA HS Assay Kit (Invitrogen) and the size and quantity of the captured library were assessed by Bioanalyzer 2100 instrument (Agilent). The sequencing results were then aligned to the *BRCA1* (NM_007294.3) and *BRCA2* (NM_000059.3) reference sequences for mutation detection using the Burrows-Wheeler Alignment tool, which further recalibrated by the Genome Analysis Toolkit (GATK) and annotated by ANNOVAR (http://www.openbioinformatics.org/annovar/). Classification of variants was performed according to ACMG criteria [17]. Benign variant and variants of uncertain significance were excluded in our study. All the pathogenic mutations detected by next generation sequencing assay were validated via Sanger sequencing on the ABI 3730XL platform (Life Technologies), which was described in our previous study [18].

### Statistical analysis

The chi-square tests or Fisher’s exact tests for categorical variables and the Mann-Whitney U tests for continuous variables were used to analyze the differences in clinicopathological characteristics between deleterious variants carriers and non-carriers. Univariate and multivariate logistic regression analysis were utilized to assess the association between clinicopathological characteristics and carrying a deleterious variants. The nomogram and artificial neural network algorithms were conducted using the R library, rms (6.3-0) and RSNNS (0.4–14) package, individually [19]. To evaluate the feasibility and performance of the artificial neural network, a conventional multivariate logistic regression model was also constructed for comparison. The logistic regression model was developed using factors selected by univariate analysis as well. The predictive accuracy of the two models were estimated by receiver operating characteristic (ROC). Comparison of ROC curves was carried out using the method described by DeLong et al. [20]. SAS software, version 9.4 (SAS Institute), IBM SPSS Statistics 22.0 software (IBM Corporation) and the R version 4.1 software (The R Foundation for Statistical Computing) were used in this study [21–23]. A *P* value of < 0.05 was adopted as statistical significance.

## Results

### Clinicopathological characteristics of bilateral breast cancer patients

Among the 123 BBC patients, the clinicopathological characteristics of deleterious variants carriers and non-carriers are summarized in Table 1. When diagnosed as primary breast cancer for the first time, BRCA deleterious variants carriers were younger than non-carriers, median age 41.5 years vs. 48.0 years (*P* = 0.0003) respectively. We also performed statistical analysis and found that the interval when the proportion of metachronous bilateral breast cancer patients among the BRCA carriers was significantly higher than in the non-carriers from 2 years to 5 years. Furthermore, 45.0% (9/20) of the BRCA deleterious variants carriers were diagnosed with at least one triple negative breast cancer (TNBC) tumor, 15.0% (3/20) with at least one medullary carcinoma, 25.0% (5/20) with both tumors’ hormone receptor negative, 70.0% (14/20) both *HER2* negative tumors and 20.0% (4/20) both TNBC, which was higher than that in non-carriers respectively. However, menophania, menopausal status and family history did not differ significantly between patients with and without *BRCA1/2* deleterious variants. In brief, deleterious variant carriers were more likely to be hormone receptor negative, *HER2* negative breast cancer, or medullary carcinoma positive.

**Table 1:** Clinical-pathological characteristics of deleterious variants carriers and non-carriers

Characteristics	BRCA carriers	Non-carriers	***P***
Median age at first diagnosis (year)	41.5 (28–57)	48.0 (31–85)	0.0003
Median menophania (year)	15.0 (12–19)	15.0 (11–20)	0.833
Menopausal status	4/20 (20.0%)	32/96 (33.3%)	0.241
Median lactation (month)	8.5 (0–32)	10 (0–48)	0.358
Family history	4/20 (20.0%)	12/103 (11.7%)	0.310
Median interval time (year)	5.2 (0-17.4)	2.1 (0-27.8)	0.393
Interval time ≥ 1y	16/20 (80.0%)	57/103 (55.3%)	0.040
Interval time ≥ 2y	16/20 (80.0%)	52/103 (50.5%)	0.015
Interval time ≥ 3y	15/20 (75.0%)	49/103 (47.6%)	0.025
Interval time ≥ 4y	13/20 (65.0%)	36/103 (35.0%)	0.012
Interval time ≥ 5y	12/20 (60.0%)	30/103 (29.1%)	0.008
Interval time ≥ 6y	8/20 (40.0%)	25/103 (24.3%)	0.146
Interval time ≥ 7y	6/20 (30.0%)	21/103 (20.4%)	0.342
Interval time ≥ 8y	3/20 (15.0%)	19/103 (18.4%)	0.713
Interval time ≥ 9y	2/20 (10.0%)	16/103 (15.5%)	0.522
First tumor size ≥ T2	10/18 (55.6%)	55/94 (58.5%)	0.816
First tumor nodal status negative	11/18 (61.1%)	43/93 (46.2%)	0.248
Second tumor size ≥ T2	6/18 (33.3%)	38/103 (36.9%)	0.772
Second tumor nodal status negative	12/20 (60.0%)	68/102 (66.7%)	0.566
Same histologic type in both tumors	11/18 (61.1%)	64/89 (71.9%)	0.361
Same HR status in both tumors	14/19 (73.7%)	55/84 (65.5%)	0.492
Same HER2 status in both tumors	14/14 (100%)	41/62 (66.1%)	0.011
At least one HR (-) tumor	10/20 (50.0%)	39/101 (38.6%)	0.343
At least one HER2 (-) tumor	19/20 (95.0%)	82/100 (82.0%)	0.146
At least one TNBC	9/20 (45.0%)	16/100 (16.0%)	0.004
At least one medullary carcinoma	3/20 (15.0%)	1/103 (1.0%)	0.001
Both HR (-) tumors	5/20 (25.0%)	6/103 (5.8%)	0.006
Both HER2 (-) tumors	14/20 (70.0%)	33/103 (32.0%)	0.001
Both TNBCs	4/20 (20.0%)	0/101 (0.0%)	<0.0001

### The prevalence and spectrum of deleterious variants in ***BRCA1*** and ***BRCA2*** genes

**Fig. 1:**
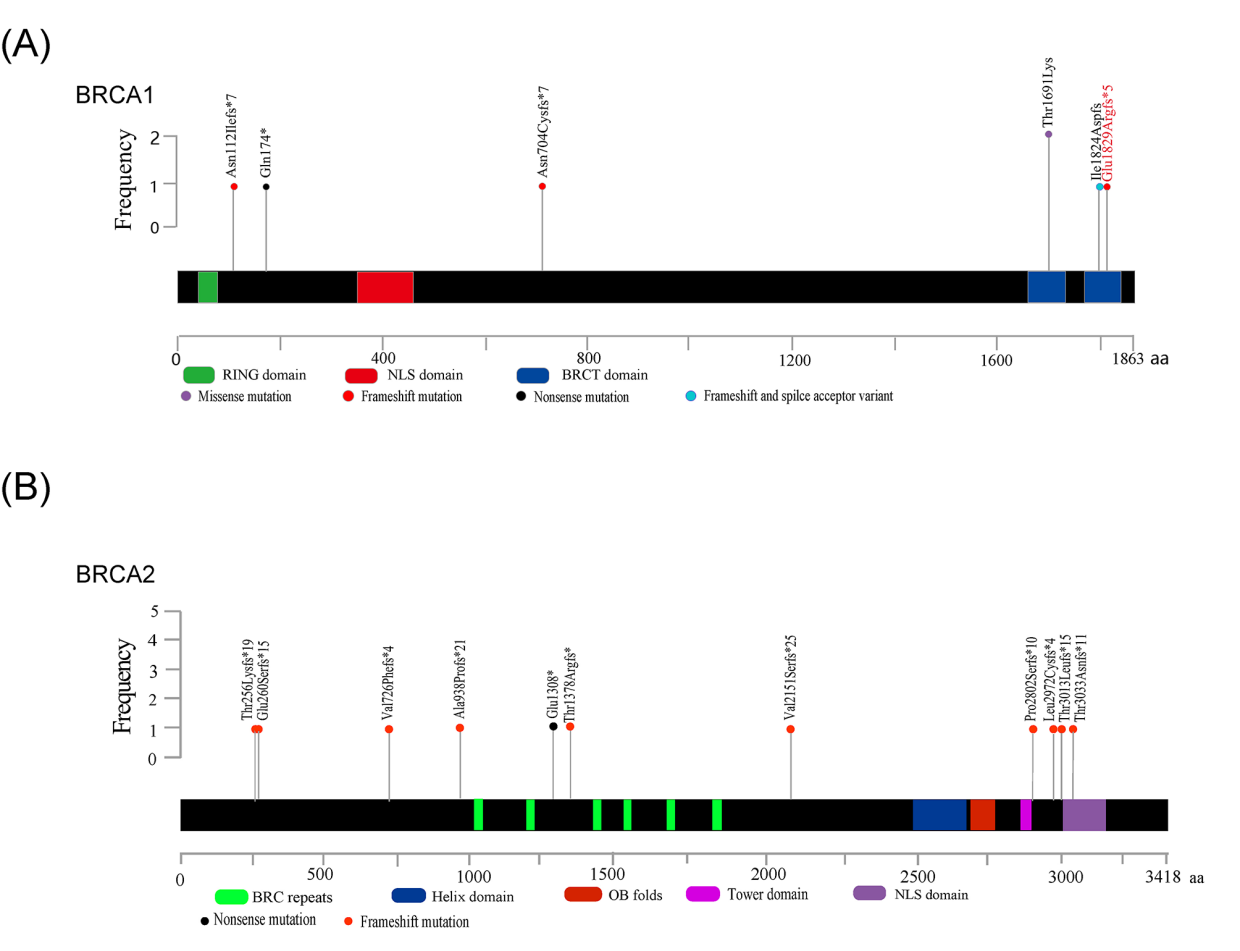
Schematic diagram of the spectrum of *BRCA1***(A)** and *BRCA2***(B)** germline mutation detected in our study. Intronic mutation rs80358163 in *BRCA1* was not shown in the schematic diagram

Of all the 123 participants, 20 patients who carried a deleterious variant were identified, eight (6.5%) in the *BRCA1* gene and 12 (9.8%) in the *BRCA2* gene (Fig. 1; Table 2). All deleterious variants were detected once except for the missense variant (c.5072 C > A) and the frameshift variant (c.9097dupA). As shown in Table 2, a total of 18 deleterious variants are listed (seven in *BRCA1* and 11 in *BRCA2*), including 13 frameshift variants (c.335delA, c.2110_2111delAA, c.5485delG, c.767_768delCA, c.774_775delAA, c.2175delA, c.2808-2811del, c.4133_4136del, c.6448dupA, c.8399_8400insA, c.8915delT, c.9037delA and c.9097dupA), two nonsense variants (c.520 C > T and c.3922G > T), one frameshift variant & splice acceptor variant (c.5470_5477delATTGGGCA), one intron variant (c.213-12 A > G), and one missense variant (c.5072 C > A). Deleterious variants, c.5485delG in *BRCA1* and c.8399_8400insA in *BRCA2* were not listed in ClinVar (https://www.ncbi.nlm.nih.gov/clinvar/) or dbSNP (http://www.ncbi.nlm.nih.gov/projects/SNP) [24, 25]. However, c.8399_8400insA in *BRCA2* was already reported [26]. All sequence variants were shown in supplementary table.

**Table 2:** Deleterious variants identified in 123 bilateral breast cancer patients of this study

Gene	Location	dbSNP Id	HGVS.c	HGVS.p	Consequence Type	Variation ID	Significance	No. of observations
*BRCA1*	Intron 4	rs80358163	c.213-12 A > G	/	Intron Variant	37,450	Pathogenic	1
*BRCA1*	Exon 6	rs886040119	c.335del	p.Asn112Ilefs*7	Frameshift	266,359	Pathogenic	1
*BRCA1*	Exon 7	rs1567806048	c.520 C > T	p.Gln174*	Nonsense	575,899	Pathogenic	1
*BRCA1*	Exon 10	rs80357814	c.2110_2111del	p.Asn704Cysfs*7	Frameshift	54,462	Pathogenic	1
*BRCA1*	Exon 16	rs80357034	c.5072 C > A	p.Thr1691Lys	Missense	37,627	Likely Pathogenic	2
*BRCA1*	Exon 23	rs80357973	c.5470_5477del	p.Ile1824Aspfs	Frameshift & splice acceptor variant	55,591	Pathogenic	1
*BRCA1*	Exon 23	Novel	c.5485del	p.Glu1829Argfs*5	Frameshift	/	Pathogenic	1
*BRCA2*	Exon 9	rs80359670	c.767_768del	p.Thr256Lysfs*19	Frameshift	52,382	Pathogenic	1
*BRCA2*	Exon 9	rs75096777	c.774_775del	p.Glu260Serfs*15	Frameshift	188,425	Pathogenic	1
*BRCA2*	Exon 11	rs276174819	c.2175del	p.Val726Phefs*4	Frameshift	926,187	Pathogenic	1
*BRCA2*	Exon 11	rs80359351	c.2808_2811del	p.Ala938Profs*21	Frameshift	9,322	Pathogenic	1
*BRCA2*	Exon 11	rs80358638	c.3922G > T	p.Glu1308*	Nonsense	37,867	Pathogenic	1
*BRCA2*	Exon 11	rs80359430	c.4133_4136del	p.Thr1378Argfs*	Frameshift	51,602	Likely Pathogenic	1
*BRCA2*	Exon 11	rs80359594	c.6448dup	p.Val2151Serfs*25	Frameshift	52,107	Pathogenic	1
*BRCA2*	Exon 19	/	c.8399_8400insA	p.Pro2802Serfs*10	Frameshift	/	Pathogenic	1
*BRCA2*	Exon 22	rs397508019	c.8915del	p.Leu2972Cysfs*4	Frameshift	52,701	Pathogenic	1
*BRCA2*	Exon 23	/	c.9037del	p.Thr3013Leufs*15	Frameshift	1,454,156	Pathogenic	1
*BRCA2*	Exon 23	rs397507419	c.9097dup	p.Thr3033Asnfs*11	Frameshift	38,208	Pathogenic	2

### Univariate logistic regression analysis for bilateral breast cancer patients

Fifteen clinicopathological characteristics factors were analyzed in a univariate analysis. We show in Table 3 that the deleterious variant status was significantly correlated with six factors including age at the first diagnosis (*P* = 0.0012), interval between diagnosis of the two tumors (*P* = 0.0211), at least one TNBC (*P* = 0.0056), at least one medullary carcinoma (*P* = 0.0146), both tumors HR negative (*P* = 0.0114) and both tumors *HER2* negative (*P* = 0.0026). TNBC is one specific type of breast cancer by lacking of the expression of estrogen receptor (ER), progesterone receptor (PR) and human epidermal growth factor receptor-2 (HER2), which exists multicollinearity in HR (-) and HER2 (-). Therefore, the variable “at least one TNBC” was not used in further analysis.

**Table 3:** Univariable logistic regression analysis between clinical-pathological characteristics and *BRCA1/2* mutations

Characteristics	OR	95% CI	***P***
Age (year)			
> 45	Reference		
≤ 45	6.088	2.042–18.149	0.0012
Menophania (year)			
> 13	Reference		
≤ 13	1.750	0.627–4.884	0.2853
Menopausal status			
Postmenopausal	Reference		
Pre-menopausal	0.500	0.154–1.619	0.2476
Lactation time (month)			
> 10	Reference		
≤ 10	1.922	0.669–5.521	0.225
Family history			
negative	Reference		
positive	1.896	0.543–6.618	0.3159
Interval time (year)			
< 2	Reference		
≥ 2	3.922	1.228–12.532	0.0211
First tumor size			
≤T2	Reference		
≥T2	0.886	0.321–2.449	0.8160
First tumor nodal status			
positive	Reference		
negative	1.827	0.651–5.126	0.2521
Second tumor size			
≤T2	Reference		
≥T2	0.855	0.297–2.465	0.772
Second tumor nodal status			
positive	Reference		
negative	0.750	0.280–2.008	0.567
Same histologic type in both tumors			
no	Reference		
yes	0.614	0.214–1.762	0.3643
Same HR status in both tumor			
no	Reference		
yes	1.476	0.484–4.505	0.4939
Same HER2 status in both tumor			
no	Reference		
yes	> 999.999	< 0.001->999.999	0.9534
At least one HR (-) tumor			
no	Reference		
yes	1.590	0.607–4.168	0.3457
At least one HER2 (-) tumor			
no	Reference		
yes	4.171	0.524–33.204	0.1773
At least one TNBC			
no	Reference		
yes	4.296	1.533–12.038	0.0056
At least one medullary carcinoma			
no	Reference		
yes	18.000	1.768-183.287	0.0146
Both HR (-) tumor			
no	Reference		
yes	5.389	1.461–19.883	0.0114
Both HER2 (-) tumor			
no	Reference		
yes	4.949	1.746–14.030	0.0026
Both TNBC			
no	Reference		
yes	> 999.999	< 0.001->999.999	0.9707

### Model development and comparison for predicting the risk of **BRCA** deleterious variants in BBC

**Fig. 2:**
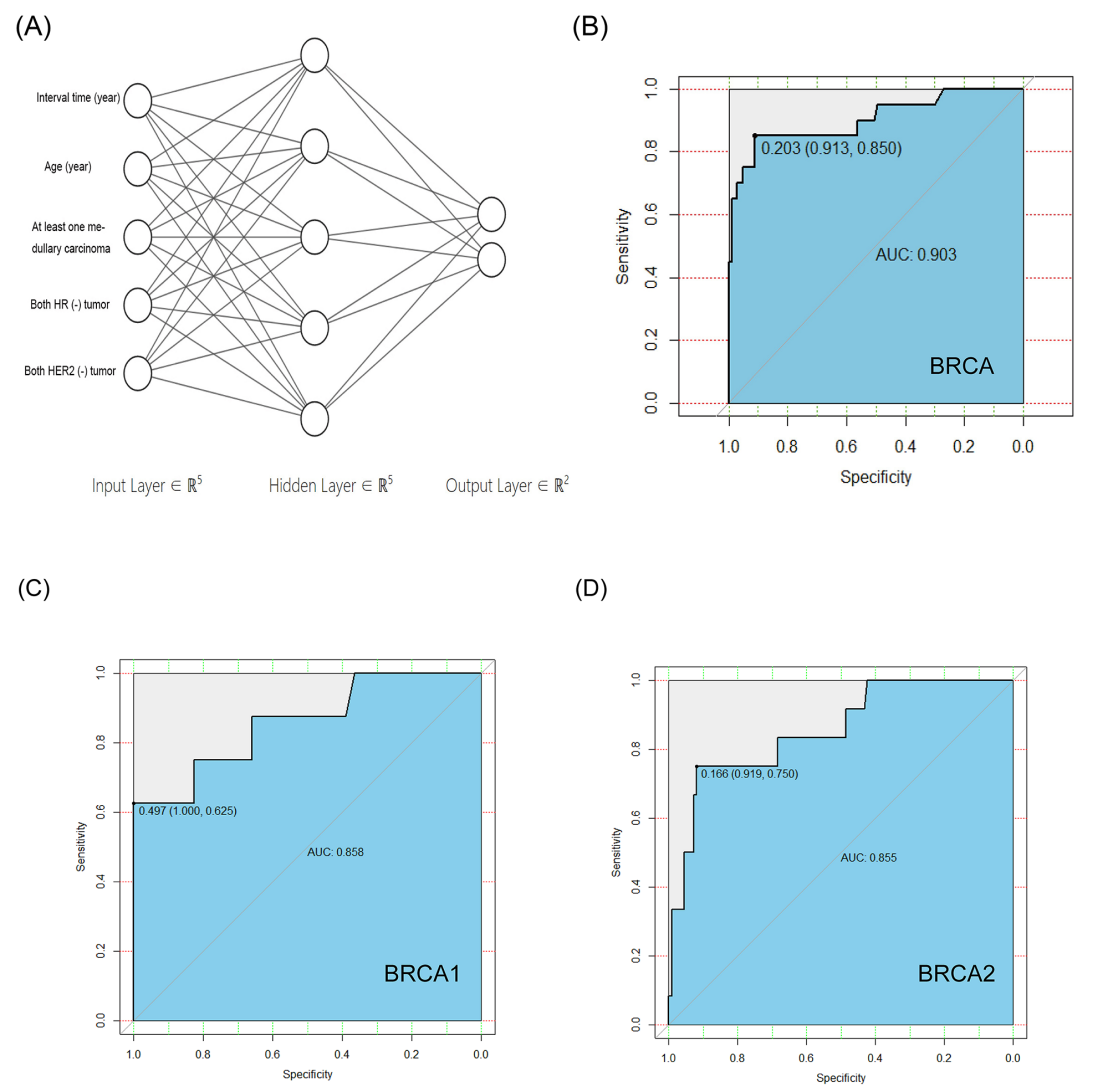
ANN for predicting the risk of *BRCA1/2* germline mutation in Chinese bilateral breast cancer patients. **(A)** Three layers of the ANN model. The accuracy of ANN in BRCA **(B)**, *BRCA1***(C)** and *BRCA2***(D)** germline mutation prediction are assessed by ROC curve

To keep raw data as much as possible, variables identified by univariate analysis were regarded as continuous variables as possible and incorporated to build the artificial neural network model. An input layer composed by five neurons mentioned above and the hidden layer was with 5 neurons (Fig. 2 A). A multivariate analysis was subsequently performed in Table 4 and these predictors were together included to build the logistic regression model.

**Table 4:** Multivariable logistic regression analysis between clinical histopathological characteristics and *BRCA1/2* mutations

Characteristics	Effect	S.E.	OR	95% CI	*P*
Interval time (year)	-0.0209	0.0543	0.979	0.88–1.089	0.6998
Age (year)	-0.1415	0.0497	0.868	0.787–0.957	0.0045
At least one medullary carcinoma	2.6974	1.6657	14.841	0.56704–388.41	0.1054
Both HR (-) tumor	1.2346	1.0064	3.4369	0.47807–24.708	0.2199
Both HER2 (-) tumor	1.7107	0.62778	5.533	1.6166–18.938	0.0064

**Fig. 3:**
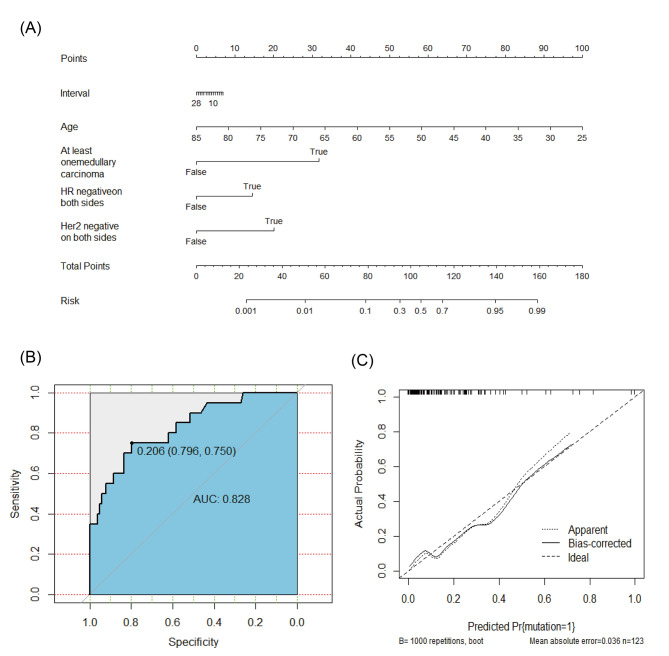
Nomogram for predicting the risk of *BRCA1/2* germline mutation in Chinese bilateral breast cancer patients. **(A)** Nomogram model. **(B)** The accuracy of nomogram in *BRCA1/2* germline mutation prediction is assessed by ROC curve, which shows good performance (Area under curve, 0.828). **(C)** The calibration curve of the nomogram in predicting *BRCA1/2* germline mutation

**Fig. 4:**
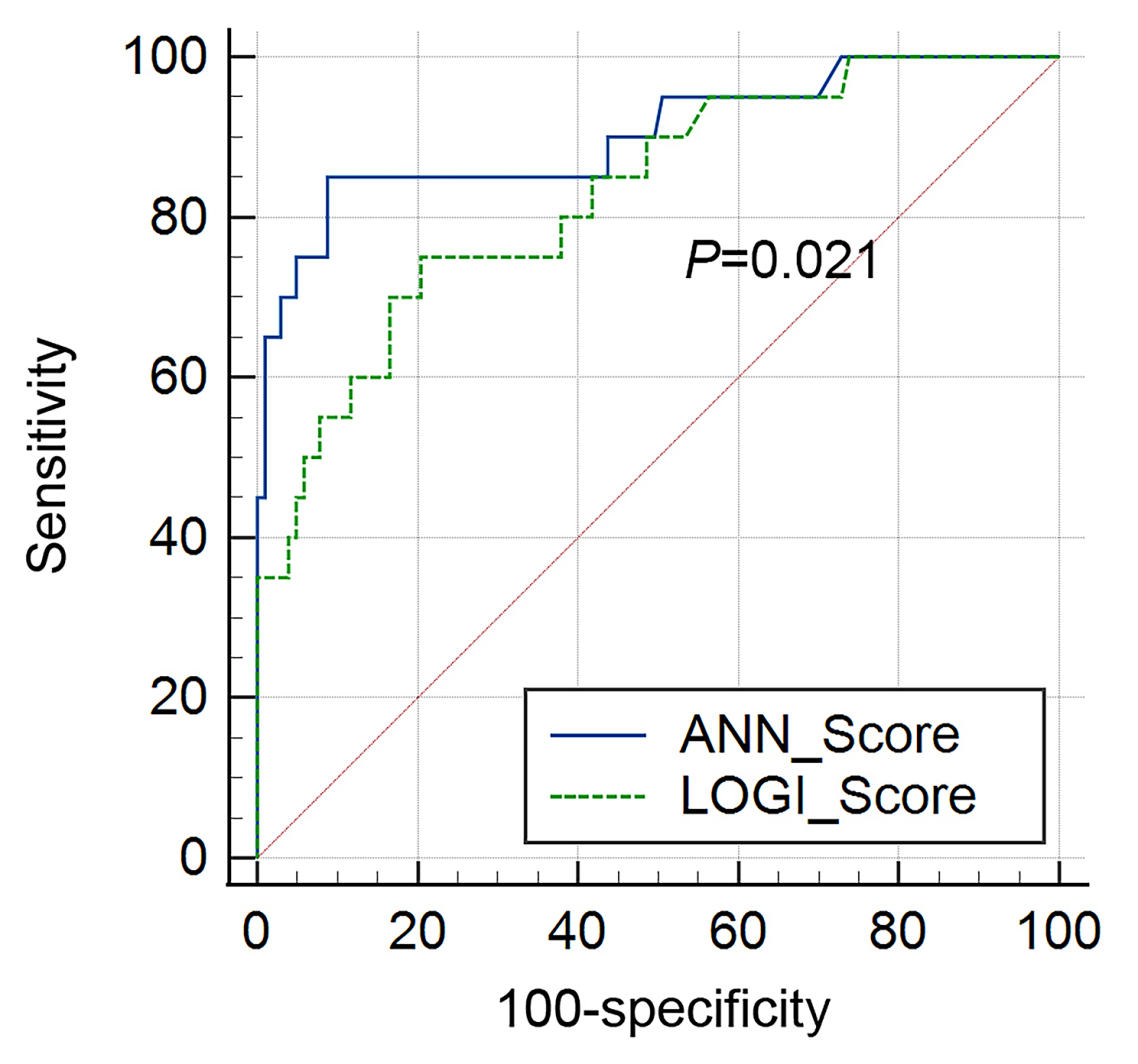
Comparison of ROC curve between the artificial neural network and logistic regression models

The AUC for the artificial neural network model (Fig. 2B-D) was 0.903 (95% C.I. = 0.836–0.949, 0.858 for *BRCA1* and 0.855 for *BRCA2*), while the AUC for nomogram predicting the risk of *BRCA1/2* germline deleterious mutation in Chinese bilateral breast cancer patients was 0.828 (95% C.I. = 0.750–0.890, Fig. 3). Predictive performance of the artificial neural network was superior to that of the logistic regression model (*P* = 0.021) (Fig. 4). We applied a cutoff of 0.203 for artificial neural network and achieved a sensitivity of 91.3% and a specificity of 85.0%, while logistic regression nomogram model on *BRCA1* and *BRCA2* separately showed excellent predictive performance in *BRCA1* (AUC 0.929) in supplementary Fig. 1 and Fig. 2.

## Discussion

In our study, variables identified in univariate analysis were incorporated within the modeling design for predicting BRCA deleterious variants in BBC patients in China, including age at the first diagnosis, interval between the two tumors, at least one TNBC, at least one medullary carcinoma, both tumors HR negative and both tumors *HER2* negative. Our ANN model showed an effective capability for outcome prediction with an AUC of 0.903. Besides, the artificial neural network model is more accurate than the logistic regression model (AUC = 0.828, *P* = 0.021). These findings show the feasibility and validity for predicting BRCA deleterious variant in BBC patients in China. To our knowledge, this is the first study of the *BRCA1/2* deleterious variant spectrum and characteristics in Chinese BBC patients and/or reporting model to predict the risk of deleterious variant status, which showed great potential application value in health economics.

*BRCA1/2* deleterious variants were detected in 20 of 123 BBC patients (16.3%) in our study. The frequency was higher than in unselected breast cancer patients (5.4%) [9], while not significantly differed from other BBC reports from China, 16.3% vs. 23.3%, 12.2% and 12.5%, respectively [9, 27, 28]. Likewise, the prevalence of deleterious variants in our study is similar to Korean and Bulgarian BBC patients, but lower than Polish BBC patients [8, 29, 30]. Our study also found that 12 (9.8%) BBC patients carried *BRCA2* deleterious variants, higher than eight (6.5%) identified with *BRCA1* deleterious variants, which was similar to other Chinese studies that evaluated unselected breast cancer patients [27, 28, 31]. The *BRCA1* deleterious variants are more likely to occur in Caucasians, particularly in Latin American and Polish patients [8, 32]. In addition, exon 11 of the *BRCA2* gene was the most frequently mutated. This is consistent with the results of previous studies [27, 33]. No damaging variants were located in exon 11 of *BRCA1* gene. The most prevalent deleterious variant in our study was *BRCA1* c. 5470_5477del in was detected only once and *BRCA2* c. 9097dupA was detected twice while they have been frequently reported in Chinese populations [9, 28]. The *BRCA1* c.5470_5477del is founder for the Chinese Han population and is associated with poor prognosis [34]. It is very important that our study also identified novel frameshift variant, c.5485delG in *BRCA1*, which was found in first diagnosed at 28 years old as metachronous bilateral triple negative breast cancer patient with unilateral medullary carcinoma.

Patients with two breast cancer lesions, including bilateral breast cancer, are recommended for further genetic risk evaluation by NCCN Guidelines [10]. There is a multitude of well known BRCA mutation-carrier prediction models like BRCAPRO, Myriad and BOADICEA [35–37]. BRCAPRO is a Bayesian statistical mode based on data from white individuals and Myriad II is an empirical model based on the testing experience of Myriad Genetics Laboratorie, while both of them underpredicted Asian carriers by two-fold and showed less accurate discrimination between Asian carriers and noncarriers. BOADICEA incorporating genetic and nongenetic risk factors is also with the AUC of 0.73 [13–16]. Chinese clinicians face serious difficulties in estimating the probability of patients carrying a deleterious variant for the propose of conducting genetic risk evaluation for those high-risk patients, especially BBC patients. What’s more, in the whole world, few models can perform well in confirming the *BRCA1/2* deleterious variant carriers in BBC patients at the lowest cost [38].

ANN have been successfully applied to address a variety of clinical problems, which provides a powerful and accurate predictive method superior to traditional statistical methods [39–41]. Previously, several studies have confirmed the potential of artificial neural network models in predicting gene deleterious variants, including *EGFR* and *BRAF* [31, 42, 43]. We collected a variety of easily accessible clinical characteristics and developed easy-to-use tool for decision-making by physicians and patients, which not add any costly and time-consuming upfront testing procedures. Therefore, an ANN model may be widely applied and popularized, especially in developing countries.

Our study also has some limitations. First, the number of our BBC patients is small, and they derived from a single center in China. Therefore, this model is not appropriate for all situations. A larger scale study and further external validation are required to fully determine its universal utility. Second, the model simply provides an assessment of individual risk for *BRCA1/2* deleterious variants while specific treatment recommendations are not offered. Future targeted research projects should address these limitations.

## Conclusion

In our retrospective study of 123 BBC cases, the spectrum of the *BRCA1/2* germline deleterious variant in Chinese BBC patients was well elaborated. This study shows and provides effective ANN modeling for predicting the risk of a *BRCA1/2* deleterious variant in BBC patients without adding any additional examinations.

## Electronic supplementary material

Below is the link to the electronic supplementary material.

Supplementary Material 1

Supplementary Material 2

Supplementary Material 3

## Data Availability

The datasets used and/or analysed during the current study are available in the BioProject repository [PRJNA859288].
