# Supplementary figures and images for: Clinical application of artificial neural network (ANN) modeling to predict BRCA1/2 germline deleterious variants in Chinese bilateral primary breast cancer patients
Source: BMC Cancer. 2022 Nov 2;22:1125. doi: 10.1186/s12885-022-10160-y (PMC9628090; doi:10.1186/s12885-022-10160-y)

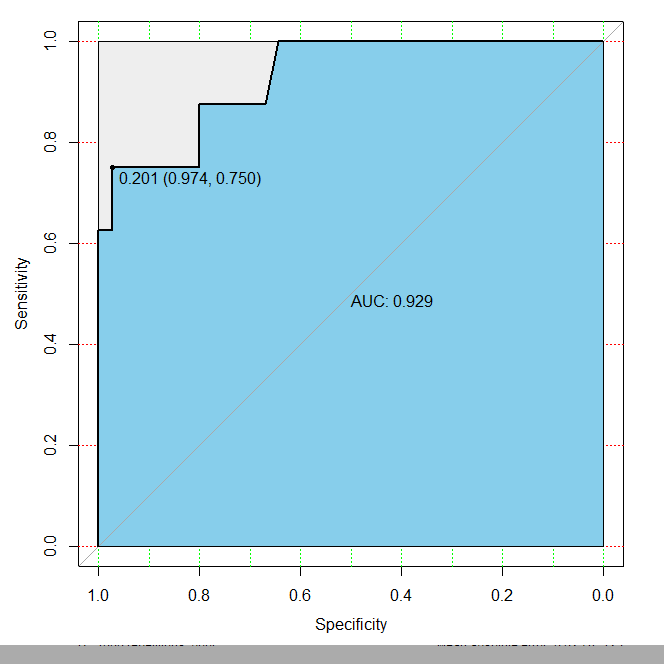

Supplement: Supplementary file 2 — Supplementary Material 2 [file 12885_2022_10160_MOESM2_ESM.png]

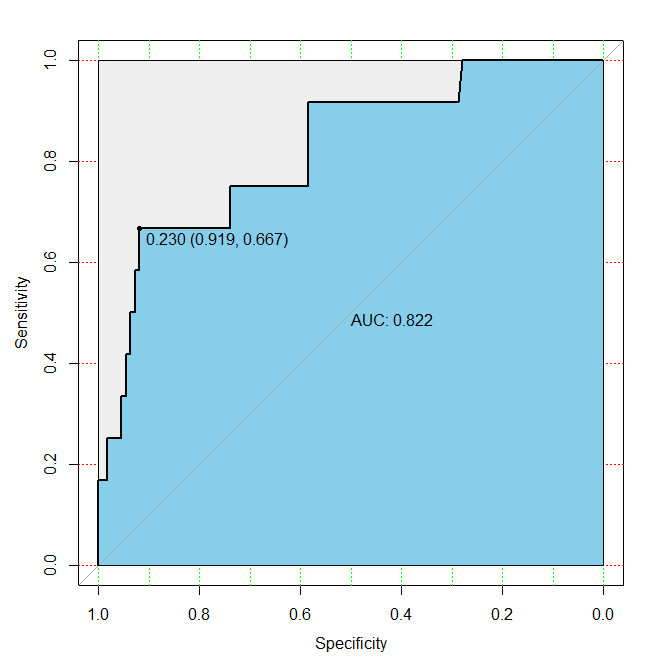

Supplement: Supplementary file 3 — Supplementary Material 3 [file 12885_2022_10160_MOESM3_ESM.png]
